# Interactive Effects of Temperature and Packaging on NDMA, DMA, and TMAO in Roasted Alaska Pollock Fillets and Pathway Regulation

**DOI:** 10.3390/foods15142537

**Published:** 2026-07-17

**Authors:** Zhuozhen Qian, Junan Pan, Jiaying Su, Shuifen Tang, Yifen Chen, Xiaoyan Wei, Zhiyu Liu, Fang Luo, Zhenyu Lin

**Affiliations:** 1 Key Laboratory of Cultivation and High-Value Utilization of Marine Organisms in Fujian Province, Fisheries Research Institute of Fujian, 7 Haishan Road, Xiamen 361013, China; 2 Fujian Jiarong Food Co., Ltd., No. 888 Nanbin Avenue, Zhangzhou Development Zone, Zhangzhou 363105, China; 3 Ministry of Education Key Laboratory of Analytical Science for Food Safety and Biology, Fujian Provincial Key Laboratory of Analysis and Detection for Food Safety, College of Chemistry, Fuzhou University, Fuzhou 350116, China; 4 College of Biological Science and Engineering, Fuzhou University, Fuzhou 350116, China

**Keywords:** *N*-nitrosodimethylamine (NDMA), dimethylamine (DMA), trimethylamine *N*-oxide (TMAO), roasted Alaska pollock fillets, storage temperature, packaging

## Abstract

To elucidate the synergistic effects of storage temperature and packaging on *N*-nitrosodimethylamine (NDMA) and its precursors dimethylamine (DMA) and trimethylamine *N*-oxide (TMAO) in roasted Alaska pollock fillets, two storage experiments were conducted. In Experiment I, commercially packaged fillets were stored at −20, 4, 10, 20, and 30 °C for 310 d. A two-way factorial ANOVA revealed a significant temperature × time interaction (*F*(24, 70) = 39.386, *p* < 0.001, partial *η*^2^ = 0.931). NDMA formation was delayed until day 172 at −20 °C; however, the 4, 10, and 20 °C groups exceeded the 4 μg/kg limit by day 263, with the 20 °C group peaking at day 263 (4.26 μg/kg) before declining to 3.73 μg/kg by day 310. In Experiment II, a three-way factorial ANOVA was applied to samples stored under oxygen-absorber or vacuum packaging at refrigerated (4 °C) or ambient (22 °C) temperature for 270 days. The temperature × packaging × time interaction was significant for NDMA, DMA, and TMAO (*p* < 0.001). The partial *η*^2^ for NDMA (0.933) was markedly larger than for DMA (0.540) and TMAO (0.607), indicating stronger combined effects on NDMA. The main effect of temperature on DMA was dominant (partial *η*^2^ = 0.994), whereas NDMA formation depended strongly on both temperature and packaging. During the 270-day storage, all refrigerated groups remained below the 4 μg/kg limit throughout; however, the ambient vacuum-packaged (BCZ) group transiently exceeded the limit (reaching 4.21 μg/kg on day 60) and subsequently fluctuated near the limit. These findings provide a scientific basis for designing safer storage and packaging strategies for such dried aquatic products.

## 1. Introduction

Alaska pollock (*Gadus chalcogrammus*), belonging to the order Gadiformes, family Gadidae, and genus *Gadus*, is a high-value demersal fish inhabiting cold waters [1]. Processed products such as fillets, salted products, and fish oil are favoured by consumers due to their high content of high-quality protein, polyunsaturated fatty acids, and various minerals [2,3]. These nutrients play a positive role in promoting neurodevelopment, maintaining cardiovascular health, and mediating anti-inflammatory and immunomodulatory functions [4,5]. However, trimethylamine *N*-oxide (TMAO), which naturally occurs in the muscle of Alaska pollock, can be degraded by endogenous enzymes or microorganisms, producing trimethylamine (TMA) and dimethylamine (DMA), respectively [6]. TMA, primarily generated by microbial TMAO reductase, has been widely used as a key chemical spoilage indicator for aquatic products [7]. DMA formation exhibits significant species specificity and is commonly observed in gadoid fish and some cephalopods during frozen storage and processing [8]. Among the formation mechanisms of various *N*-nitrosamines, DMA is recognized as the critical precursor amine for NDMA [9].

*N*-nitrosamines are potent carcinogens formed through the nitrosation of secondary amines with nitrite under weakly acidic conditions [10]. They can be classified into volatile and non-volatile *N*-nitrosamines based on molecular weight and vapour pressure [11]. As a representative volatile *N*-nitrosamine, NDMA is classified as a Group 2A carcinogen by the International Agency for Research on Cancer (IARC) due to its significant hepatotoxicity and carcinogenicity [12]. According to the Chinese national food safety standard GB 2762-2022 [13], the maximum allowable limit for NDMA in aquatic products (excluding canned aquatic products) is 4 μg/kg. NDMA formation is influenced by multiple factors, including heat treatment temperature, pH, and microbial community structure [14,15,16,17]. Additionally, lipid oxidation products generated during high-temperature frying, such as malondialdehyde and other reactive aldehydes, can accelerate NDMA formation [18,19]. Long-term excessive dietary intake of NDMA can lead to liver fibrosis and multi-organ damage, posing a persistent health risk to humans [20]. Notably, NDMA accumulation in roasted Alaska pollock fillets is closely governed by processing conditions [21,22], yet it is equally affected by post-processing storage factors, including time, temperature, and packaging method [23,24,25]. Despite this dual dependency, existing research has largely focused on the monitoring of terminal NDMA residues and exposure risk assessment of dried aquatic products [26,27], leaving the dynamic evolution patterns throughout the entire storage period largely unexplored. Admittedly, a previous study has reported NDMA occurrence in roasted Alaska pollock fillets during processing and storage [24]; however, that work did not systematically evaluate NDMA dynamics across a wide temperature gradient over an extended timeframe, nor did it integrate long-term temperature assessment with packaging-related monitoring of DMA and TMAO. Consequently, a comprehensive understanding of the combined effects of storage temperature, duration, and packaging on NDMA evolution remains lacking.

Focusing on the post-processing storage stage, this study aims to: (1) systematically investigate the formation patterns and dynamic characteristics of NDMA across a range of storage temperatures over a 310-day period; (2) systematically evaluate the synergistic effects of packaging methods (commercial packaging, oxygen-absorber packaging, vacuum packaging) and storage temperatures (ambient, refrigerated) on the contents of NDMA and its precursors DMA, TMA, and TMAO over a 9-month storage period using non-suppressed ion chromatography and liquid chromatography-tandem mass spectrometry (LC-MS/MS), and (3) elucidate the variation patterns and intrinsic correlations among NDMA and its precursor amines during storage, assess the inhibitory effects of different packaging and temperature combinations on NDMA formation, and identify the optimal storage and packaging strategy. The findings will provide a theoretical basis and data support for the safe storage and packaging design of dried aquatic products such as roasted Alaska pollock fillets.

## 2. Materials and Methods

### 2.1. Chemicals and Reagents

Trimethylamine *N*-oxide dihydrate (purity ≥ 99%) and trimethylamine hydrochloride standards (purity ≥ 98%) were purchased from Sigma-Aldrich, St. Louis, MO, USA. Dimethylamine standard (purity ≥ 98%) was purchased from Macklin Biochemical Technology Co., Ltd., Shanghai, China. NDMA (1000 μg/mL) and NDMA-D6 (1000 μg/mL) standard solution were supplied by Manhage Biotechnology Co., Ltd., Shanghai, China. Methanesulfonic acid (purity 99.9%) and isooctane (chromatographic grade) were from Macklin Biochemical Technology Co., Ltd., Shanghai, China. Trichloroacetic acid (purity 99%), sulfuric acid, sodium chloride (high-purity grade), and anhydrous sodium sulfate (chromatographic grade) were from Xilong Scientific Co., Ltd., Guangzhou, China. Chloroform and dichloromethane (chromatographic grade) were obtained from Sinopharm Chemical Reagent Co., Ltd., Shanghai, China. Ultrapure water (18.2 MΩ·cm) was used throughout the experiments.

### 2.2. Preparation of Standard Solutions

Standard stock solutions of TMAO, TMA, and DMA (500 mg/L, calculated as free amines) were prepared by weighing appropriate amounts of trimethylamine oxide dihydrate, trimethylamine hydrochloride, and dimethylamine hydrochloride, dissolving them in ultrapure water, and diluting to 100 mL. These solutions were stored in sealed containers at 4 °C in the dark.

NDMA standard solution and NDMA-D6 internal standard solution (both 1 μg/mL) were prepared by diluting 1 mL of the respective 1000 μg/mL standards to 1000 mL with dichloromethane and stored at 4 °C in the dark.

### 2.3. Storage Experiments

Samples from the same batch of roasted Alaska pollock fillets were homogenized prior to packaging and storage to minimize initial compositional heterogeneity and ensure internal comparability among treatment groups. The homogenized samples (20.0 g each) were then subjected to three methods: commercial packaging (food-grade PE ziplock bag, provided by Fujian Jiarong Food Co., Ltd., which is the actual packaging used for the commercially available product), oxygen-absorber packaging (commercial packaging with an oxygen absorber sachet), and vacuum packaging. Sample identifiers were as follows: CP for commercial packaging; BLPT for oxygen-absorber packaging under refrigerated conditions; BLZ for vacuum packaging under refrigerated conditions; BCPT for oxygen-absorber packaging under ambient conditions; BCZ for vacuum packaging under ambient conditions. The moisture content of the homogenized samples was determined to be 14–16% (*w*/*w*). At each time point, three independent packages per treatment were used as biological replicates (*n* = 3) and destructive sampling was employed (i.e., separate packages were opened at each time point). For instrumental analysis, each independent sample was measured in duplicate, and the arithmetic mean of the two readings was used as the representative value for that package. It should be noted that the use of homogenized material, while necessary for experimental consistency, does not fully replicate the intact tissue structure of commercial fillet products. The results therefore represent a conservative estimate of NDMA formation, as homogenization increases the surface area exposed to oxygen.

Two sets of storage experiments were conducted.

Experiment I: To investigate the effect of storage temperature on NDMA content in commercially packaged roasted Alaska pollock fillets, samples were stored at −20, 4, 10, 20, and 30 °C. NDMA content was determined on days 0, 30, 60, 90, 172, 263, and 310, with day 0 serving as the control.

Experiment II was designed to further explore the synergistic effects of packaging method and storage temperature on NDMA and its precursor amines, based on the results of Experiment I. Since Experiment I confirmed that commercial packaging allowed NDMA to exceed the regulatory limit (4 μg/kg) under storage conditions above 4 °C, commercial packaging was deemed unsuitable for long-term storage (270 days) and was therefore excluded from Experiment II. Instead, oxygen-absorber-packaged and vacuum-packaged samples were stored at refrigerated (4 °C) and ambient (22 ± 1 °C) temperatures, simulating retail storage conditions. NDMA, DMA, TMA, and TMAO contents were measured on days 0, 15, 30, 60, 90, 120, 150, 180, 210, 240, and 270, with day 0 as the control. It is acknowledged that this design limits the direct comparison of oxygen-absorber and vacuum packaging with the original commercial packaging within the same experiment.

### 2.4. Sample Pretreatment

#### 2.4.1. Measurements of DMA, TMA, and TMAO

In a 50 mL centrifuge tube, 0.2 g of homogenized sample was combined with 2 mL of chloroform and 5 mL of 1% (*v*/*v*) trichloroacetic acid solution pre-cooled to 4 °C. After vortexing, the mixture was kept at 4 °C for 20 min, vortexed at 3000 rpm for 30 s, and immediately sonicated in a pre-cooled (12 °C) ultrasonic bath for 10 min. Subsequently, the sample was centrifuged at 6000 r/min for 5 min at 4 °C, after which the supernatant was collected. The precipitate was re-extracted with 5 mL of pre-cooled 1% trichloroacetic acid solution, and the two supernatants were combined. An aliquot (2 mL) of the combined extract was then centrifuged at 10,000 r/min for 10 min at 4 °C and filtered through a 0.22-μm hydrophilic membrane for ion chromatography analysis.

#### 2.4.2. Measurements of NDMA

A homogenized sample (20.0 g) was placed in a distillation flask, and 40 μL of NDMA-D6 internal standard intermediate solution, 100 mL of ultrapure water, and 50 g of sodium chloride were added sequentially. For collection, 50 mL of dichloromethane and 0.5 mL of isooctane were added to the receiving flask, with the condenser outlet positioned below the liquid surface and cooling maintained using an ice–water bath. After distillation for 8 min, 200–250 mL of distillate was collected. Subsequently, 15 g of sodium chloride and 2 mL of sulfuric acid solution were added to the distillate, then stirred until dissolved, and transferred to a separatory funnel. The mixture was shaken at 230 rpm for 5 min, and then the dichloromethane layer was collected. The aqueous phase was subsequently extracted three additional times with 120 mL of dichloromethane. The combined dichloromethane extracts (approximately 170 mL) were dehydrated over 10 g of anhydrous sodium sulfate and concentrated to 5–10 mL using a rotary evaporator in a 20 °C water bath. The concentrate was further concentrated to 0.8 mL under a gentle stream of nitrogen, transferred to a 1.0 mL volumetric flask, and brought to volume with dichloromethane. The extract was filtered through a 0.22-μm organic-phase membrane for LC-MS/MS analysis.

### 2.5. Instrumental Analysis Conditions

#### 2.5.1. Ion Chromatography Conditions

Ion chromatography separation was performed using a Thermo Fisher Scientific ICS-600 ion chromatography system (Waltham, MA, USA) equipped with a Dionex IonPac CS17 column (4 × 250 mm) (Sunnyvale, CA, USA). The mobile phase was 3.08 mmol/L methanesulfonic acid solution at a flow rate of 0.8 mL/min. The column temperature was 15 °C, and the injection volume was 10 μL. A non-suppressed conductivity detector was used, with a data acquisition frequency of 5.0 Hz.

#### 2.5.2. Liquid Chromatography–Mass Spectrometry Conditions

A TSQ Altis Plus liquid chromatograph-mass spectrometer (Thermo Fisher Scientific Waltham, MA, USA) equipped with a CNW Athena C18 column (3 μm, 4.6 × 150 mm) (ANPEL Laboratory Technologies (Shanghai) Inc., Shanghai, China) was used for separation. The LC conditions were 40 °C column temperature, 0.6 mL/min flow rate, and 5 μL injection volume. Mobile phase A was 0.1% formic acid, and mobile phase B was methanol. The gradient programme was 0–4 min, a decrease in A from 92% to 5%; 4–5 min, 5% A maintained; 5–6 min, an increase in A from 5% to 92%; 6–11 min, 92% A maintained. MS conditions were atmospheric pressure chemical ionization (APCI) source, sheath gas flow 45 Arb, auxiliary gas flow 10 Arb, and sweep gas flow rate of 2 Arb. The ion transfer tube temperature was 300 °C, vaporizer temperature was 400 °C, and selected reaction monitoring (SRM) mode was used. For NDMA, the precursor ion was 75.1, and the product ions were 43.1 and 58.1; for NDMA-D6, the precursor ion was 81.0 and the product ion was 46.0.

### 2.6. Analytical Method Validation

The analytical methods were validated with respect to the following parameters: calibration ranges, linearity (correlation coefficients, R^2^), limit of detection (LOD), limit of quantification (LOQ), recovery (spiked at three concentration levels), intra-day and inter-day precision (relative standard deviation, RSD), and matrix effect (for LC-MS/MS). Detailed validation data are provided in Supplementary Table S1. All concentrations are expressed on a dry-weight basis.

### 2.7. Data Processing and Statistical Analysis

All experiments were performed in three independent biological replicates (*n* = 3), and results are expressed as mean ± standard deviation (SD). Prior to ANOVA, Levene’s test was used to assess the homogeneity of variances across groups. Experiment I employed a two-way between-subjects factorial ANOVA with storage temperature (5 levels) and storage time (7 levels) as fixed factors. Experiment II employed a three-way between-subjects factorial ANOVA with storage temperature (2 levels), packaging method (2 levels) and storage time (11 levels) as fixed factors, testing all main effects and interactions. When a significant interaction was detected, simple effects analysis was conducted with Bonferroni correction for multiple comparisons. Polynomial contrasts were applied to the time factor to evaluate linear, quadratic, and cubic trends of the dependent variables over the storage period. All statistical analyses were performed using SPSS 19 (IBM Corp., Armonk, NY, USA). A *p*-value < 0.05 was considered statistically significant. Partial *η*^2^ values are reported as measures of effect size.

## 3. Results and Discussion

### 3.1. Variation in NDMA Content in Commercially Packaged Roasted Alaska Pollock Fillets During Storage

**Figure 1:**
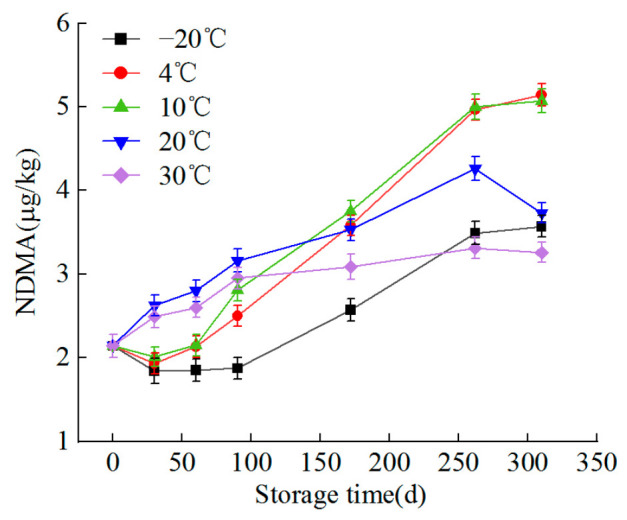
Dynamic changes in NDMA content in commercially packaged roasted Alaska pollock fillets under different storage temperatures. Data are shown as mean ± SD (*n* = 3).

Experiment I examined the dynamic changes in NDMA content in commercially packaged roasted Alaska pollock fillets stored at different temperatures. The results are presented in Figure 1. A two-way between-subjects factorial ANOVA revealed that the time × temperature interaction effect was statistically significant (*F*(24, 70) = 39.386, *p* < 0.001, partial *η*^2^ = 0.931), indicating that the patterns of change in NDMA content over time differed significantly among the temperature groups [28,29]. The temperature main effect (*F*(4, 70) = 134.538, *p* < 0.001, partial *η*^2^ = 0.885) and the time main effect (*F*(6, 70) = 697.992, *p* < 0.001, partial *η*^2^ = 0.984) were also significant. Levene’s test confirmed homogeneity of variances across groups (*F*(34, 70) = 0.029, *p* = 1.000).

As shown in Figure 1, during the initial storage period (0–30 d), NDMA content showed a decreasing trend in the −20, 4, and 10 °C groups, whereas an increasing trend was observed in the 20 and 30 °C groups. Polynomial contrast analysis revealed significant linear, quadratic, and cubic trends for NDMA over time (all *p* < 0.001; overall time effect: *F*(6, 70) = 697.992, *p* < 0.001, partial *η*^2^ = 0.984), indicating a complex, non-monotonic accumulation pattern across the storage period. Simple effects analysis further statistically confirmed these differences: at day 30, the NDMA content in the 20 °C group was significantly higher than its initial level (mean difference = 0.48 μg/kg, *p* = 0.017), while the increase in the 30 °C group was not statistically significant (*p* = 0.142), indicating that the nitrosation reaction was initiated more rapidly at 20 °C.

During mid-storage (30–263 d), NDMA content continued to increase across all temperature groups, but the accumulation rates exhibited clear temperature dependence. In the −20 °C group, NDMA content showed no significant change during the first 90 d of storage (*p* > 0.05), and a significant increase was first observed on day 172, with a mean difference of 0.43 μg/kg (*p* = 0.030), confirming that freezing effectively delays the NDMA formation window. The 4 and 10 °C groups displayed similar patterns: NDMA content was relatively stable during 30–60 d but significantly increased from day 90 onward. Compared to day 30, the mean differences were 0.57 μg/kg (*p* < 0.001) at 4 °C and 0.797 μg/kg (*p* < 0.001) at 10 °C, with a larger increase at 10 °C, demonstrating that elevated temperature within this range accelerates early NDMA accumulation. By day 172, the formation rates in the 4 and 10 °C groups surpassed those in the −20, 20, and 30 °C groups. By day 263, NDMA content in the 4, 10, and 20 °C groups all exceeded the Chinese national standard limit of 4 μg/kg (GB 2762-2022) [13]. Notably, the NDMA content in the 10 °C group at day 263 was significantly higher than at day 172 (increase = 1.25 μg/kg, *p* < 0.001), whereas the corresponding increase in the 20 °C group was only 0.73 μg/kg (*p* = 0.001), statistically confirming the sustained high-efficiency accumulation of NDMA in the moderate temperature range.

During late storage (263–310 d), temperature effects further diverged. NDMA content in the 20 °C group decreased to 3.73 μg/kg, while the −20, 4, and 10 °C groups maintained an upward trend. Simple effects analysis provided direct statistical evidence: NDMA content in the 20 °C group decreased significantly by 0.530 μg/kg from day 263 to day 310 (*p* = 0.004), whereas no significant differences were observed among time points from day 172 to day 310 in the 30 °C group (*p* > 0.05), suggesting (as a hypothesis) that NDMA formation and loss reached a dynamic equilibrium at 30 °C, with content remaining at a relatively stable plateau.

Throughout the entire storage period, NDMA content in the 20 °C group was consistently higher than that in the 30 °C group. We hypothesize that although 30 °C facilitates the nitrosation reaction rate, it may simultaneously accelerate the chemical degradation or physical volatilization of NDMA, resulting in lower net accumulation. In contrast, the 20 °C environment might be more conducive to microbial proliferation and endogenous enzyme activity, facilitating the sustained supply of precursor amines and thereby maintaining a higher NDMA formation potential. The stagnation of NDMA content in the 30 °C group during mid-to-late storage is consistent with, but does not directly prove, the hypothesis of dual effects of high temperature—enhanced formation being offset by simultaneously enhanced degradation or volatilization.

### 3.2. Variation in NDMA, DMA, TMA, and TMAO in Different Packaging Groups During Storage

#### 3.2.1. Synergistic Effects of Packaging Method and Storage Temperature on NDMA Content

**Figure 2:**
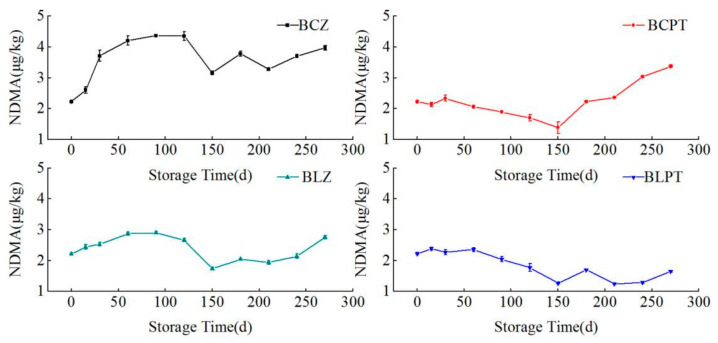
Changes in NDMA content over storage time in different packaging treatment groups. Data are shown as mean ± SD (*n* = 3).

The trends in NDMA content for each treatment group in Experiment II are shown in Figure 2. A three-way between-subjects factorial ANOVA revealed a statistically significant three-way interaction of temperature × packaging × time (*F*(10, 88) = 121.633, *p* < 0.001, partial *η*^2^ = 0.933), indicating that the inhibitory effect of packaging on NDMA varies depending on storage temperature and storage stage [30]. The temperature main effect (*F*(1, 88) = 5274.284, *p* < 0.001, partial *η*^2^ = 0.984), packaging main effect (*F*(1, 88) = 7115.329, *p* < 0.001, partial *η*^2^ = 0.988), and time main effect (*F*(10, 88) = 304.752, *p* < 0.001, partial *η*^2^ = 0.972) were all significant. The two-way interactions were also significant: temperature × packaging (*F*(1, 88) = 1254.350, *p* < 0.001, partial *η*^2^ = 0.934), temperature × time (*F*(10, 88) =220.162, *p* < 0.001, partial *η*^2^ = 0.962), and packaging × time (*F*(10, 88) = 207.267, *p* < 0.001, partial *η*^2^ = 0.959). Polynomial contrast analysis revealed significant linear, quadratic, and cubic trends for NDMA over time (all *p* < 0.001), with the cubic component showing the largest estimate (0.829), reflecting the complex multiphasic accumulation–decline–re-accumulation pattern of NDMA across storage stages.

Under ambient temperature conditions, the NDMA content in the BCZ group increased rapidly during the early storage period, reached 4.21 μg/kg on day 60, and subsequently fluctuated around 4.0 μg/kg, ending at 3.97 μg/kg on day 270 (a 78.03% increase from the initial value). This group represented the highest cumulative risk among all treatment groups. The transient exceedance of the regulatory limit (4 μg/kg) indicates that ambient vacuum packaging could not effectively halt the continuous progression of nitrosation. The NDMA content in the BCPT group displayed a clear pattern of an initial decrease followed by an increase: starting from an initial value of 2.23 μg/kg, it declined to 1.39 μg/kg on day 150 (the lowest value over the entire period), then gradually recovered, rising to 3.38 μg/kg on day 270, representing a 51.57% increase relative to the initial value. This U-shaped trajectory, consistent with the significant quadratic component of the three-way interaction, suggests that the oxygen absorber may have exerted inhibitory effects during the early-to-mid storage period (0–150 d), reducing NDMA content by approximately 37.67% from the initial level. The subsequent increase in NDMA after day 150 could be related to the gradual depletion of oxygen-scavenging capacity, although this interpretation remains speculative in the absence of direct measurements of residual oxygen levels or oxygen-absorber activity. Alternatively, other factors not measured in this study, such as changes in nitrite/nitrate concentrations, pH, or redox potential, may also contribute to the accelerated NDMA formation during the later storage stage, and these possibilities warrant further investigation. Based on the above data, a safe storage period of no more than 150 days is recommended for ambient-temperature oxygen-absorber-packaged products.

Under refrigerated conditions, NDMA content in all packaging groups exhibited limited variation. The BLZ group showed a slight increase of 23.32% over 270 days, while the BLPT group displayed an overall slow decreasing trend, with the final content lower than the initial level. These data consistently indicate that low-temperature storage is considerably more effective than packaging method in inhibiting NDMA formation under the conditions tested.

#### 3.2.2. Synergistic Effects of Packaging Method and Storage Temperature on DMA Content

**Figure 3:**
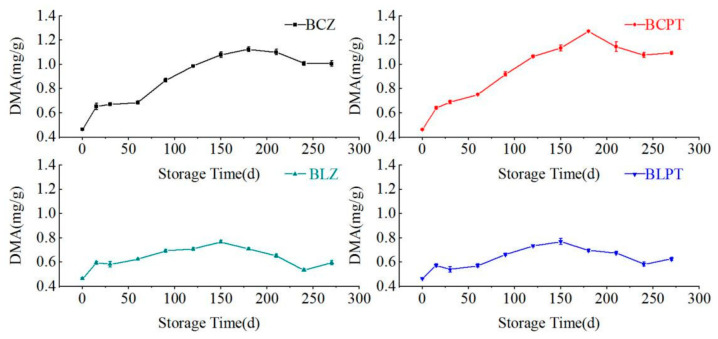
Changes in DMA content over storage time in different packaging treatment groups. Data are shown as mean ± SD (*n* = 3).

The DMA content trends in Experiment II are shown in Figure 3. Although DMA itself has not yet been incorporated into food safety limit standards, as the key precursor amine for NDMA formation, its accumulation level during storage is of great significance for risk assessment of subsequent nitrosation [31,32]. A three-way between-subjects factorial ANOVA showed a significant three-way interaction of temperature × packaging × time for DMA (*F*(10, 88) = 10.315, *p* < 0.001, partial *η*^2^ = 0.540). The temperature main effect (*F*(1, 88) = 14,704.215, *p* < 0.001, partial *η*^2^ = 0.994) and time main effect (*F*(10, 88) = 1696.811, *p* < 0.001, partial *η*^2^ = 0.995) both showed extremely large effect sizes, whereas the packaging main effect (*F*(1, 88) = 140.531, *p* < 0.001, partial *η*^2^ = 0.615) was considerably smaller. The temperature × time interaction (*F*(10, 88) = 574.213, *p* < 0.001, partial *η*^2^ = 0.985) was also much stronger than the packaging × time interaction (*F*(10, 88) = 17.022, *p* < 0.001, partial *η*^2^ = 0.659), indicating that temperature plays a more important role than packaging in modulating DMA accumulation over time. Polynomial contrast analysis revealed significant linear, quadratic, and cubic trends for DMA over time(all *p* < 0.001; overall time effect: *F*(10, 88) = 1696.811, *p* < 0.001, partial *η*^2^ = 0.995), indicating that DMA accumulation is predominantly linear with secondary curvature reflecting rate changes across storage stages.

Under ambient temperature conditions, DMA content in both packaging groups exhibited a continuous, nearly linear increase. In the BCZ group, DMA increased from 0.47 mg/g to 1.01 mg/g (114.89% increase). The accumulation in the BCPT group was even more pronounced, rising from 0.47 mg/g to 1.10 mg/g (134.04% increase). Notably, the DMA content in the BCPT group had already approached 1.0 mg/g by day 90 (0.92 mg/g), peaked at 1.28 mg/g on day 180, and thereafter remained around 1.10 mg/g. The sustained high-level accumulation of DMA in this group coincided temporally with the rebound of NDMA after day 150—DMA had already reached a high plateau of 1.14 mg/g by day 150, whereas NDMA just began to accelerate its formation after day 150 (rebounding from 1.39 μg/kg to 3.38 μg/kg by day 270). This temporal offset is consistent with the mechanistic inference that DMA accumulation is a necessary prerequisite for NDMA formation, but its conversion to NDMA is regulated by redox conditions—a hypothesis that requires direct confirmation in future studies.

Under refrigerated conditions, DMA accumulation was significantly inhibited. The DMA content in the BLZ group slowly increased from 0.47 mg/g to 0.60 mg/g (a 27.66% increase), representing only 24.07% of that in the corresponding ambient group. The BLPT group increased from 0.47 mg/g to 0.63 mg/g (a 34.04% increase), representing 25.40% of the ambient counterpart. DMA contents in both refrigerated groups did not exceed 0.77 mg/g, and the increase was mainly concentrated in the first 90 days, after which it plateaued. These comparisons indicate that low temperature effectively controls the DMA formation rate and restricts the NDMA formation scale at the substrate supply level. The much larger effect size for temperature (partial *η*^2^ = 0.994) compared with packaging (partial *η*^2^ = 0.615) further supports the conclusion that at the regulatory node of DMA formation, temperature is the dominant factor, while packaging exerts only a limited modulating effect.

#### 3.2.3. Effect of Packaging on TMA Content During Storage

The limit of detection (LOD) for TMA in this study was 0.21 mg/g (equivalent to 210 mg/kg). Throughout the 270-day storage period, TMA levels in all treatment groups remained consistently below this threshold and could not be reliably quantified. This result is consistent with the tracking data from the preceding processing stage, in which TMA became undetectable after seasoning and high-temperature processing and did not re-accumulate during subsequent storage under the conditions tested. Based on these results, TMA does not appear to be a major precursor contributing to NDMA formation in this study system; however, this conclusion is constrained by the sensitivity of the employed analytical method. More sensitive techniques (e.g., LC-MS/MS) would be required to definitively assess trace-level TMA accumulation. Therefore, the subsequent correlation analysis will focus on the two metabolic pathways involving DMA and TMAO.

#### 3.2.4. Synergistic Effects of Packaging Method and Storage Temperature on TMAO Content

**Figure 4:**
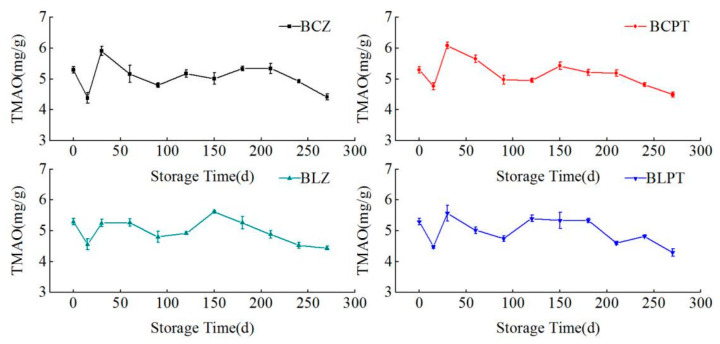
Changes in TMAO content over storage time in different packaging treatment groups. Data are shown as mean ± SD (*n* = 3).

TMAO content trends in Experiment II are shown in Figure 4. Although TMAO itself is not subject to direct regulatory limits, its degradation product DMA has been confirmed as a key precursor of NDMA; therefore, the metabolic dynamics of TMAO during storage have upstream regulatory significance for NDMA formation potential [33,34,35]. A three-way between-subjects factorial ANOVA revealed a significant three-way temperature × packaging × time interaction for TMAO (*F*(10, 88) = 13.593, *p* < 0.001, partial *η*^2^ = 0.607), indicating that the effect of packaging on TMAO degradation varied with storage temperature. The temperature main effect (*F*(1, 88) = 52.305, *p* < 0.001, partial *η*^2^ = 0.373), packaging main effect (*F*(1, 88) = 8.446, *p* = 0.005, partial *η*^2^ = 0.088), and time main effect (*F*(10, 88) = 162.886, *p* < 0.001, partial *η*^2^ = 0.949) were all significant. The temperature × time interaction (*F*(10, 88) = 17.432, *p* < 0.001, partial *η*^2^ = 0.665) was stronger than the packaging × time interaction (*F*(10, 88) = 4.008, *p* < 0.001, partial *η*^2^ = 0.313), indicating that temperature plays a more important role than packaging in modulating TMAO degradation over time. However, the three-way interaction effect size (partial *η*^2^ = 0.607) was lower than that for NDMA (partial *η*^2^ = 0.933), and the time × packaging interaction effect size was only 0.313 (much lower than 0.959 for NDMA), indicating that the regulatory capacity of packaging on TMAO degradation is much weaker than its direct regulation of NDMA formation. Polynomial contrast analysis revealed significant linear, quadratic, and cubic trends for TMAO over time (all *p* < 0.001; overall time effect: *F*(10, 88) = 162.886, *p* < 0.001, partial *η*^2^ = 0.949), reflecting the characteristic “decline–rebound–fluctuating decline” pattern across the storage period. Notably, the TMAO change curves across all groups followed a similar overall pattern, suggesting that temperature and packaging primarily affected the amplitude and timing of TMAO fluctuations rather than the fundamental shape of the curves.

Under ambient temperature conditions, TMAO content in both BCZ and BCPT groups peaked on day 30 of storage, and thereafter declined with fluctuations. By day 270, the TMAO content had decreased by 0.88 mg/g and 0.80 mg/g from the initial values, representing reductions of 16.60% and 15.09%, respectively. Under refrigerated conditions, TMAO changes in the BLZ and BLPT groups were relatively moderate, with decreases of 16.23% and 18.87% from their initial values by day 270. From a temporal perspective, the decrease, rebound, and subsequent decline fluctuation pattern of TMAO in all treatment groups stands in stark contrast to the “monotonic increase” pattern of DMA—TMAO rebounded to a peak as early as day 30, whereas DMA continued to accumulate until reaching a plateau at day 150. This suggests that DMA released from TMAO degradation is not the sole source of DMA generation, and TMAO degradation is not the only rate-limiting step affecting NDMA formation.

It is noteworthy that the TMAO content in the BLPT group was lower than that in the BCPT group at most sampling time points during the early and late storage periods, although this pattern was not entirely consistent across the entire storage duration. This observation, which contradicts the general expectation that “low temperature inhibits degradation,” is statistically supported by the significant three-way interaction (partial *η*^2^ = 0.607). A possible explanation, which requires direct experimental verification, is that the rate of the oxygen-scavenging reaction is positively correlated with temperature. Under ambient conditions, the oxygen absorber in the BCPT group may exhibit higher activity, enabling the packaging system to enter a low-oxygen state quickly, thereby terminating the oxygen-dependent non-enzymatic TMAO degradation pathway at an early stage. Under refrigerated conditions, the activity of the oxygen absorber in the BLPT group might be inhibited by low temperature, slowing the oxygen-scavenging rate and prolonging the retention of residual oxygen, which provides a wider time window for the non-enzymatic oxidative degradation of TMAO. Simultaneously, trimethylamine-*N*-oxide demethylase (TMAOase) may retain a certain level of catalytic activity under refrigerated conditions, which could also contribute to TMAO consumption [34,35]. The superposition of both non-enzymatic and enzymatic pathways in the refrigerated oxygen-absorber system could result in a greater net consumption of TMAO in the BLPT group during long-term storage. This analysis suggests a significant interaction between the oxygen scavenging kinetics of the oxygen absorber and storage temperature, indicating that packaging strategies require systematic synergistic consideration rather than simple addition.

### 3.3. Dynamic Correlation Analysis Between Amines and NDMA During Storage

**Figure 5:**
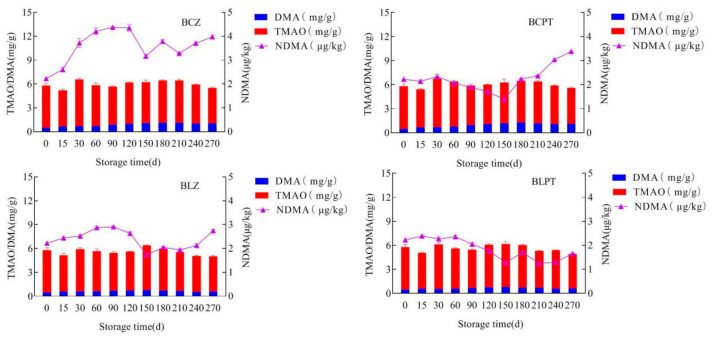
Dynamic transformation relationships between NDMA and its precursors (DMA and TMAO) in differently packaged roasted Alaska pollock fillets during storage.

Figure 5 presents the dynamic transformation relationships between NDMA and its precursor amines DMA and TMAO during storage. Integrating the above three-way ANOVA results, NDMA formation in this study system is consistent with the TMAO→DMA→NDMA reaction pathway, with the two key steps differentially regulated by distinct environmental factors [36]. To quantitatively assess the regulatory factors of the two steps in the TMAO→DMA→NDMA pathway, three-way ANOVAs were performed with DMA and NDMA contents as dependent variables, respectively, using their respective effect sizes as quantitative indicators of regulatory intensity at the corresponding nodes.

Based on the temporal correspondence between DMA and NDMA contents, the two exhibit a distinct “accumulation–lagged conversion” pattern. Under ambient conditions, DMA content in the BCPT group increased linearly from 0 to 150 d, reaching a high plateau of 1.14 mg/g by day 150, while NDMA content continuously decreased over the same period to the lowest value of the entire storage period (1.39 μg/kg). Thereafter, with DMA persistently maintained at levels above 1.10 mg/g, NDMA content rebounded rapidly from day 150 onward, rising to 3.38 μg/kg by day 270. In the BCZ group, DMA also increased throughout the entire period, with an increase of 114.89%, while NDMA increased synchronously and linearly with an increase of 78.03%, showing no lag phenomenon. This temporal offset in the BCPT group, in contrast to the synchronous relationship in the BCZ group, combined with the U-shaped curve of NDMA in the BCPT group, is consistent with the interpretation that DMA accumulation is a necessary prerequisite for NDMA formation, but the efficiency of its conversion to NDMA may be tightly regulated by the redox conditions of the system—in particular, oxygen concentration—a mechanistic hypothesis that requires direct confirmation in future studies. Statistical analysis provides quantitative support: the effect sizes of the three-way interaction (partial *η*^2^ = 0.933) and the time × packaging interaction (partial *η*^2^ = 0.959) for NDMA were both substantially higher than the corresponding values for DMA (partial *η*^2^ = 0.540 and 0.659, respectively), suggesting that the packaging method (oxygen absorber) exerts its core inhibitory effect primarily by blocking the DMA→NDMA nitrosation step, while its regulatory capacity over the TMAO→DMA degradation step is limited.

As the direct metabolic precursor of DMA, the degradation dynamics of TMAO and its “source–sink” relationship with DMA formation exhibited a more complex asynchrony. In all four treatment groups, TMAO content declined sharply and synchronously from day 0 to day 15, with an average decrease of approximately 0.75 mg/g; it then rebounded substantially to a peak from day 15 to day 30, surpassing the initial level by about 0.41 mg/g; thereafter, it declined with repeated fluctuations, yielding a net decrease of only 16–19% by day 270. In contrast, DMA content increased monotonically throughout the same period, displaying a completely divergent pattern. This observation suggests that simple degradation of TMAO is not the sole source of DMA formation, and points to possible involvement of other precursor conversion pathways and the existence of a regeneration or release mechanism for TMAO during the early stage of storage. More notably, the TMAO content in the BLPT group was significantly lower than that in the BCPT group at most time points during the early and late storage periods, suggesting that refrigerated storage coupled with oxygen-absorber packaging may have paradoxically exacerbated the net consumption of TMAO. A possible explanation for this observation, as previously described, is hypothesized to stem from the temperature dependence of the oxygen scavenging kinetics of the oxygen absorber: under ambient conditions, the high activity of the oxygen absorber rapidly removes oxygen, thereby terminating the non-enzymatic TMAO degradation pathway at an early stage; under refrigerated conditions, the oxygen absorber activity might be inhibited by low temperature, prolonging the retention of residual oxygen, while TMAOase may remain enzymatically active under such conditions. The additive effect of the enzymatic and non-enzymatic pathways in the refrigerated oxygen-absorber system could result in a greater net consumption of TMAO. However, in the absence of direct measurements of residual oxygen levels, oxygen-absorber activity, TMAOase activity, and other relevant variables (e.g., nitrite/nitrate, pH, redox potential), these mechanistic interpretations remain speculative and warrant further investigation.

## 4. Conclusions

This study systematically investigated the synergistic effects of storage temperature, packaging method, and storage time on NDMA and its precursors in roasted Alaska pollock fillets, and quantitatively revealed the differential characteristics of the two regulatory nodes in the TMAO→DMA→NDMA pathway. The results indicate that low temperature is the core factor inhibiting DMA formation, whereas the oxygen absorber appears to play a key role primarily by blocking the DMA→NDMA nitrosation step; the regulatory intensity of the two factors differs fundamentally. Refrigerated storage combined with vacuum or oxygen-absorber packaging effectively kept the NDMA content below the regulatory limit throughout the 270-day storage period. By contrast, under ambient conditions, vacuum packaging failed to prevent transient exceedance of the limit, while oxygen-absorber packaging maintained NDMA below the limit throughout the 270-day storage, a rising trend after 150 days suggests that 150 days be taken as a conservative safe storage period under ambient conditions. However, refrigerated storage significantly increases cold chain costs during commercial distribution, imposing considerable economic pressure on small- and medium-sized aquatic product processing enterprises. Therefore, future research could further explore alternative strategies under ambient temperature conditions, such as high-barrier composite packaging, modified atmosphere packaging, and the combined application of natural antioxidant coatings with packaging, to balance food safety with economic feasibility.

Several limitations of this study should be acknowledged. First, the use of homogenized samples, while ensuring internal uniformity, may have accelerated oxidation and NDMA formation relative to intact fillets; thus, the results may overestimate formation rates under real-world conditions. Second, water activity—a key parameter controlling enzymatic and oxidative reactions in dried products—was not measured. Third, the absence of direct measurements of nitrite/nitrate, pH, redox potential, and oxygen-absorber residual capacity limits the mechanistic interpretation of the observed trends. Future studies using intact fillets and incorporating these key physicochemical parameters are already being planned to validate and extend the present findings.

## Data Availability

The original contributions presented in this study are included in the article. Further inquiries can be directed to the corresponding author.

## Supplementary Materials

The following supporting information can be downloaded at: https://www.mdpi.com/article/10.3390/foods15142537/s1, Table S1: Analytical method validation parameters for NDMA, DMA, TMA, and TMAO; Table S2: Experimental I NDMA content (μg/kg) in commercially packaged roasted Alaska pollock fillets during storage at different temperatures; Table S3: Experimental II NDMA content (μg/kg) in roasted Alaska pollock fillets under different packaging and storage temperature conditions; Table S4: Experimental II DMA content (mg/g) in roasted Alaska pollock fillets under different packaging and storage temperature conditions; Table S5: Experimental II TMAO content (mg/g) in roasted Alaska pollock fillets under different packaging and storage temperature conditions.
