# Supplementary material for: Interactive Effects of Temperature and Packaging on NDMA, DMA, and TMAO in Roasted Alaska Pollock Fillets and Pathway Regulation
Source: Foods. 2026 Jul 17;15(14):2537. doi: 10.3390/foods15142537 (PMC13409537; doi:10.3390/foods15142537)
Supplement: Supplementary file 1 [file foods-15-02537-s001.zip › foods-4394021-supplementary.pdf]

foods

Supplementary File

Interactive Effects of Temperature and Packaging on NDMA, DMA, and TMAO in Roasted Alaska Pollock Fillets and the Pathway Regulation

Zhuozhen Qian [1,*], Junan Pan [1], Jiaying Su [1], Shuifen Tang [1], Yifen Chen [2], Xiaoyan Wei [2], Zhiyu Liu [1], Fang Luo [3,4,*] and Zhenyu Lin [3]

[1] Fisheries Research Institute of Fujian, Key Laboratory of Cultivation and High-value Utilization of Marine Organisms in Fujian Province, 7 Haishan Road, Xiamen 361013, China;
[2] Fujian Jiarong Food Co., Ltd., No. 888 Nanbin Avenue, Zhangzhou Development Zone, Zhangzhou 363105, China;
[3] Ministry of Education Key Laboratory of Analytical Science for Food Safety and Biology, Fujian Provincial Key Laboratory of Analysis and Detection for Food Safety, College of Chemistry, Fuzhou University, Fuzhou 350116, China;
[4] College of Biological Science and Engineering, Fuzhou University, Fuzhou 350116, China;

* Correspondence: qianzhuozhen@126.com(Z.Q.), luofang@fzu.edu.cn(F.L.)

Table S1. Analytical method validation parameters for NDMA, DMA, TMA, and TMAO.

Analyte	Method	Calibration Range	R^2	LOD (μg/kg) (mg/g)	LOQ (μg/kg) (mg/g)	Spiked (μg/kg) (mg/g)	Intra-day (n=6)		Inter-day (n=6)		Matrix Effect (%)
							Average Recovery (%)	RSD (%)	Average Recovery (%)	RSD (%)	
NDMA *	LC-MS/MS	0.25~20 μg/kg	0.9992	0.30	1.00	1.00	91.50	7.68	86.12	6.15	5.26
						5.00	93.38	5.37	90.32	6.02	
						10.00	92.25	6.14	91.63	6.97	
DMA	Ion Chromatography	0.025~2.5 mg/g	0.9982	0.051	0.17	0.25	63.83	1.82	69.66	1.93	—
						1.00	77.24	3.53	75.74	2.19	
						2.50	72.26	1.57	71.63	1.99	
TMA	Ion Chromatography	0.025~2.5 mg/g	0.9986	0.063	0.21	0.25	81.54	6.07	90.52	5.58	—
						1.00	92.39	3.86	91.68	2.79	
						2.50	89.35	3.87	86.86	3.69	
TMAO	Ion Chromatography	0.05~5.0 mg/g	0.9988	0.075	0.25	0.50	78.86	2.32	75.52	1.52	—
						2.00	73.98	2.45	73.31	1.74	
						5.00	79.70	1.10	77.86	1.34	

Notes: LOD: limit of detection; LOQ: limit of quantification; RSD: relative standard deviation. All recovery and precision data were obtained from spiked samples at three concentration levels (low, medium, high). NDMA matrix effect was evaluated by comparing peak areas of post-extraction spiked samples with neat standards in mobile phase. All concentrations are expressed on a dry-weight basis.

* NDMA is measured in μg/kg.

Table S2. Experimental I NDMA content (μg/kg) in commercially packaged roasted Alaska pollock fillets during storage at different temperatures.

Temperature	Day0	Day30	Day60	Day90	Day172	Day263	Day310
−20 °C	2.14 ± 0.14	1.84 ± 0.15	1.85 ± 0.13	1.87 ± 0.13	2.57 ± 0.14	3.49 ± 0.14	3.57 ± 0.13
4 °C	2.14 ± 0.14	1.93 ± 0.13	2.13 ± 0.13	2.50 ± 0.12	3.58 ± 0.12	4.96 ± 0.13	5.14 ± 0.13
10 °C	2.14 ± 0.14	2.01 ± 0.12	2.15 ± 0.13	2.81 ± 0.13	3.75 ± 0.13	5.00 ± 0.15	5.07 ± 0.14
20 °C	2.14 ± 0.14	2.62 ± 0.13	2.80 ± 0.13	3.16 ± 0.14	3.53 ± 0.13	4.26 ± 0.14	3.73 ± 0.12
30 °C	2.14 ± 0.14	2.49 ± 0.13	2.60 ± 0.12	2.95 ± 0.13	3.09 ± 0.15	3.31 ± 0.13	3.26 ± 0.12

Note: Values are expressed as mean ± SD (n = 3).

Table S3. Experimental II NDMA content (μg/kg) in roasted Alaska pollock fillets under different packaging and storage temperature conditions.

Storage Condition	Packaging	Day 0	Day 15	Day 30	Day 60	Day 90	Day 120	Day 150	Day 180	Day 210	Day 240	Day 270
22±1 °C (Ambient)	Vacuum (BCZ)	2.23 ± 0.03	2.60 ± 0.04	3.71 ± 0.17	4.21 ± 0.13	4.38 ± 0.02	4.35 ± 0.13	3.16 ± 0.07	3.78 ± 0.08	3.28 ± 0.01	3.71 ± 0.04	3.97 ± 0.05
22±1 °C (Ambient)	Oxygen-absorber (BCPT)	2.23 ± 0.03	2.14 ± 0.03	2.34 ± 0.09	2.07 ± 0.02	1.90 ± 0.02	1.71 ± 0.09	1.39 ± 0.17	2.24 ± 0.02	2.37 ± 0.02	3.04 ± 0.01	3.38 ± 0.02
4 °C (Refrigerated)	Vacuum (BLZ)	2.23 ± 0.03	2.44 ± 0.05	2.52 ± 0.05	2.88 ± 0.05	2.91 ± 0.01	2.64 ± 0.06	1.74 ± 0.04	2.05 ± 0.03	1.94 ± 0.04	2.13 ± 0.07	2.75 ± 0.05
4 °C (Refrigerated)	Oxygen-absorber (BLPT)	2.23 ± 0.03	2.39 ± 0.04	2.27 ± 0.06	2.36 ± 0.06	2.05 ± 0.06	1.78 ± 0.13	1.28 ± 0.03	1.70 ± 0.02	1.25 ± 0.02	1.30 ± 0.03	1.66 ± 0.03

Note: Values are expressed as mean ± SD (n = 3).

Table S4. Experimental II DMA content (mg/g) in roasted Alaska pollock fillets under different packaging and storage temperature conditions.

Storage Condition	Packaging	Day 0	Day 15	Day 30	Day 60	Day 90	Day 120	Day 150	Day 180	Day 210	Day 240	Day 270
22±1 °C (Ambient)	Vacuum (BCZ)	0.47 ± 0.01	0.66 ± 0.03	0.68 ± 0.01	0.69 ± 0.01	0.87 ± 0.02	0.99 ± 0.01	1.08 ± 0.02	1.13 ± 0.01	1.10 ± 0.02	1.01 ± 0.01	1.01 ± 0.02
22±1 °C (Ambient)	Oxygen-absorber (BCPT)	0.47 ± 0.01	0.65 ± 0.01	0.69 ± 0.01	0.75 ± 0.01	0.92 ± 0.01	1.07 ± 0.01	1.14 ± 0.03	1.28 ± 0.00	1.15 ± 0.04	1.08 ± 0.02	1.10 ± 0.01
4 °C (Refrigerated)	Vacuum (BLZ)	0.47 ± 0.01	0.60 ± 0.01	0.58 ± 0.02	0.63 ± 0.01	0.69 ± 0.01	0.71 ± 0.00	0.77 ± 0.01	0.71 ± 0.01	0.66 ± 0.02	0.54 ± 0.01	0.60 ± 0.01
4 °C (Refrigerated)	Oxygen-absorber (BLPT)	0.47 ± 0.01	0.58 ± 0.01	0.54 ± 0.02	0.57 ± 0.01	0.66 ± 0.01	0.74 ± 0.01	0.77 ± 0.01	0.70 ± 0.01	0.68 ± 0.01	0.59 ± 0.01	0.63 ± 0.01

Note: Values are expressed as mean ± SD (n = 3).

Table S5. Experimental II TMAO content (mg/g) in roasted Alaska pollock fillets under different packaging and storage temperature conditions.

Storage Condition	Packaging	Day 0	Day 15	Day 30	Day 60	Day 90	Day 120	Day 150	Day 180	Day 210	Day 240	Day 270
22±1 °C (Ambient)	Vacuum (BCZ)	5.30 ± 0.07	4.39 ± 0.14	5.91 ± 0.16	5.17 ± 0.26	4.80 ± 0.06	5.18 ± 0.12	5.02 ± 0.14	5.34 ± 0.05	5.34 ± 0.11	4.93 ± 0.04	4.42 ± 0.07
22±1 °C (Ambient)	Oxygen-absorber (BCPT)	5.30 ± 0.07	4.77 ± 0.08	6.09 ± 0.05	5.66 ± 0.05	4.98 ± 0.11	4.96 ± 0.01	5.43 ± 0.08	5.22 ± 0.10	5.19 ± 0.04	4.81 ± 0.06	4.50 ± 0.05
4 °C (Refrigerated)	Vacuum (BLZ)	5.30 ± 0.07	4.57 ± 0.19	5.26 ± 0.03	5.27 ± 0.09	4.81 ± 0.19	4.92 ± 0.02	5.62 ± 0.03	5.26 ± 0.10	4.88 ± 0.13	4.53 ± 0.10	4.44 ± 0.02
4 °C (Refrigerated)	Oxygen-absorber (BLPT)	5.30 ± 0.07	4.48 ± 0.05	5.57 ± 0.22	5.02 ± 0.10	4.76 ± 0.06	5.40 ± 0.07	5.34 ± 0.20	5.34 ± 0.03	4.61 ± 0.02	4.83 ± 0.04	4.30 ± 0.13

Note: Values are expressed as mean ± SD (n = 3).
